# Supplementary material for: Differential Predictors of Response to Early Start Denver Model vs. Early Intensive Behavioral Intervention in Young Children with Autism Spectrum Disorder: A Systematic Review and Meta-Analysis
Source: Brain Sci. 2022 Nov 4;12(11):1499. doi: 10.3390/brainsci12111499 (PMC9688546; doi:10.3390/brainsci12111499)
Supplement: Supplementary file 1 [file brainsci-12-01499-s001.zip › Supplementary Tables.pdf]

Supplementary Information

Differential predictors of response to Early Start Denver Model vs Early Intensive Behavioral Intervention in young children with Autism Spectrum Disorder: a systematic review and meta-analysis.

Asta Lisa, Antonio M. Persico*

Child & Adolescent Neuropsychiatry Program, Modena University Hospital, & Department of Biomedical, Metabolic and Neural Sciences, University of Modena and Reggio Emilia, Modena, Italy

*Correspondence: antonio.persico@unimore.it

Table S1. Complete lists of all articles extracted from each database, distinguished by database source (sheets 1-4), and specifying the cause for exclusion (sheet 5).

Table S2. Summary of EIBI studies excluded from our systematic review due to a patient recruitment age range beyond 48 months: sample characteristics.

Study	Cases				Controls			
	N (M:F)	Age at intake in months (mean)	Diagnosis	Exclusion criteria	Control Intervention	N (M:F)	Age at intake in months (mean)	Diagnosis
[1]Dimian et al., 2021	667 (548:119)	24-72 (mean age n.r)	n.r.	n.r.	---	---	---	---
[2,3]Eikeseth et al., 2002/2007	13 (7:5)	48-84 (66)	ICD-10; ADI-R	IQ > 50; Age at intake < 48 and > 84; Severe medical conditions	Eclectic	12 (11:1)	48-84 (65)	ASD
[4]Eikeseth et al., 2012	35 (29:6)	25-76 (47)	ICD-10	Authors report excluding one child because she was re-diagnosed with Rett's syndrome diagnosis	TAU	24 (20:4)	24-88 (53)	ASD
[5]Eldevik et al., 2006	13 (10:3)	36-68 (53)	ICD-10; ADI-R	Severe medical conditions; CA > 72 at treatment start	Eclectic	15 (14:1)	21-69 (49)	ASD
[6]Eriksson et al., 2013	93 (gender n.r.)	20-54 (38)	DSM-IV	"complex" cases with ASD; no exclusion of children with medical/genetic/neurological conditions or ID	Less-intensive ABA	105 (gender n.r.)	20-54 (43,5)	ASD

Study	Cases				Controls			
	N (M:F)	Age at intake in months (mean)	Diagnosis	Exclusion criteria	Control Intervention	N (M:F)	Age at intake in months (mean)	Diagnosis
[7]Flanagan et al., 2012	79 (gender n.r.)	range n.r. (43)	CARS	No exclusion criteria based on comorbid diagnosis or skills level	None (Waitlist for IBI)	63 (gender n.r.)	range n.r. (43)	ASD
[8]Frazier et al., 2021	131 (114:17)	17-71 (40)	DSM-IV/ DSM-5 ADOS CARS	Not based on symptoms, cognitive or functional severity	---	---	---	---
[9]Goin-Kochel et al., 2007	29 (27:2)	30-61 (46)	ADI-R ADOS	Not being registered in the school where EIBI was delivered	---	---	---	---
[10]Granpeesheh et al., 2009	245 (gender n.r.)	16-144 (73)	Not reported	Age at intake < 16 or > 144 months; Being in treatment for < 1 or > 48 months	---	---	---	---
[11]Harris & Handleman, 2000	27 (23:4)	31-65 (49)	DSM-III; CARS	n.r.	---	---	---	---
[12]Klintwall and Eikeseth, 2012	21 (16:5)	27-59 (43)	CARS	Authors report excluding one child because she was re-diagnosed with Rett's syndrome diagnosis	---	---	---	---
[13]Lewon and Ghezzi 2021	13 (13:0)	28-56	DSM-IV GARS-2	Comorbid psychiatric disorder	---	---	---	---
[14]Luiselli et al., 2000	16 (15:1)	26-57 (m=32 for children aged < 36 months; m=48 for children aged > 36 months)	Clinical diagnosis independent of the study	n.r.	---	---	---	---

Study	Cases				Controls			
	**N (M:F)**	**Age at intake in months (mean)**	**Diagnosis**	**Exclusion criteria**	**Control Intervention**	**N (M:F)**	**Age at intake in months (mean)**	**Diagnos is**
[15,16]Magiati et al., 2007; 2011	28 (27:1)	22-54 (38)	Clinical diagnosis independent of the study; ADI-R	Severe medical conditions	TAU	16 (12:4)	22-54 (42.5)	ASD
[17]Perry et al., 2011	332 (276:56)	20-86 (54)	DSM-IV; CARS	No exclusion criteria based on neurological/genetic conditions	---	---	---	---
[18]Préfontaine et al., 2022	233 (184:49)	24-69 (52)	Clinical diagnosis independent of the study; CARS-2	Age beyond 60 months at intake	---	---	---	---
[19]Rivard et al., 2019	32 (21:11)	37-59	GARS-2; CARS	n.r.	---	---	---	---
[20]Smith et al., 2015	71 (60:11)	24-59 (39)	ADOS; ADI-R	Severe medical/genetic conditions	---	---	---	---
[21]Stoelb et al., 2004	19 (14:5)	26-122 (56)	DSM-IV; CARS	n.r.	---	---	---	---
[22]Virués-Ortega et al., 2013	24 (21:3)	range n.r. (50)	DSM-IV-R; ADOS-2; ADI-R	No exclusion based on children functioning	---	---	---	---
[23]Waters et al., 2018	48 (45:3)	18-75 (38)	Clinical diagnosis independent of the study; ADI-R	Severe medical conditions; IQ>35	TAU	46 (45:1)	18-75 (42)	ASD

Study	Cases				Controls			
	N (M:F)	Age at intake in months (mean)	Diagnosis	Exclusion criteria	Control Intervention	N (M:F)	Age at intake in months (mean)	Diagnos is
[24]Weiss, 1999	20 (19:1)	20-65 (41.5)	DSM-IV; CARS	n.r.	---	---	---	---

ADI-R: Autism Diagnostic Interview-Revised; ADOS: Autism Diagnostic Observation Schedule; ASD: Autism Spectrum Disorder; CARS: Childhood Autism Rating Scale; DSM: Diagnostic and Statistical Manual of Mental Disorders; ESDM: Early Start Denver Model; GARS: Gilliam Autism Rating Scale; IBI: Intensive Behavioral Intervention; ICD: International Classification of Diseases; n.r.: Not Reported; P-EIBI: Parent-delivered Early Intensive Behavioral Intervention; TAU: Treatment as Usual.

Table S3. Summary of EIBI studies excluded from our systematic review due to a patient recruitment age range beyond 48 months: interventions characteristics.

Study	Country	Study design	Interevention type	Setting	Intensity	Duration
[1]Dimian et al., 2021	USA	Retrospective population-based observational study	EIBI	n.r.	19 hrs/wk	24 months
[2,3]Eikeseth et al., 2002/2007	Norway	Case-control trial/Follow-up	UCLA EIBI	School based	28 hrs/wk	12 mo
[4]Eikeseth et al., 2012	Sweden	Comparison-controlled trial	UCLA EIBI	Preschool/Kindergarten	15-37 hrs/wk	12 mo

Study	Country	Study design	Interevention type	Setting	Intensity	Duration
[5]Eldevik et al., 2006	Norway	Retrospective study	UCLA EIBI	Kindergarten /Elementary school	12,5 hrs/wk	20 mo
[6]Eriksson et al., 2013	Sweden	Prospective population-based two-year follow-up study	EIBI	Home-based; Kindergarten (see Fernell et al. 2011)	15-40 hrs/wk	24 mo
[7]Flanagan et al., 2012	Canada	Retrospective study	IBI	Community-based; Home-based (occasionally)	26 hrs/wk	24 mo
[8]Frazier et al., 2021	USA	Retrospective study	EIBI	School based	30 hrs/wk	2-49 (m=24) mo
[9]Goin-Kochel et al., 2007	USA	Retrospective study	EIBI	Special ASD kindergarten	30 hrs/wk	2-28 (m=11) mo
[10]Granpeesheh et al., 2009	USA	Retrospective study	ABA	Community-based	20-168 (m=78)	n.r.
[11]Harris & Handleman, 2000	USA	One group pretest-posttest design	ABA	University center-based preschool program	35-45 hrs/wk	12 mo
[12]Klintwall and Eikeseth, 2012	Sweden	One group pretest-posttest design	UCLA EIBI	Home-based; Kindergarten	20 hrs/wk	12 mo
[13]Lewon and Ghezzi 2021	USA	Retrospective study	EIBI	n.r.	15-38 (m=30) hrs/wk	39 mo

Study	Country	Study design	Interevention type	Setting	Intensity	Duration
[14]Luiselli et al., 2000	USA	Retrospective study	UCLA EIBI	Home-based	12-15 hrs/wk	7-11 mo
[15,16]Magiati et al., 2007; 2011	UK	Case-control trial/Follow-up	UCLA EIBI	Home-based	32 hrs/wk	24 mo
[17]Perry et al., 2011	Canada	Retrospective study	IBI	Home-based; Center-based; Childcare setting	20-40 hrs/wk	4-47 (m=18) mo
[18]Préfontaine et al., 2022	Canada	Prospective longitudinal study	EIBI	Community-based	4-12 hrs/wk (low intensity) or 16-20 hrs/week (moderate intensity)	12 mo
[19]Rivard et al., 2019	Canada	One group pretest-posttest design/1-year follow-up	UCLA EIBI	n.r.	10-20 (m=15) hrs/wk	12 mo
[20]Smith et al., 2015	USA	One group pretest-posttest design	UCLA EIBI	Community-based	15 hours/week for 12 months	12 mo
[21]Stoelb et al., 2004	USA	Retrospective study	UCLA EIBI	Home/School/Daycare setting	12-36 hours/week for 12 months	12 mo
[22]Virués-Ortega et al., 2013	Spain	One group pretest-posttest design	UCLA EIBI	Home-based	15-47 (m=31) hrs/wk	5-59 (m=22) mo
[23]Waters et al., 2018	USA	Case-control trial	UCLA EIBI	n.r.	35-40 hrs/wk	36 mo
[24]Weiss, 1999	USA	Retrospective study	EIBI	Home-based	40 hrs/wk	24 mo

ABA: Applied Behavioral Analysis; EIBI: Early Intensive Behavioral Intervention; IBI: Intensive Behavioral Intervention; IQ: Intellectual Quotient; MA: Mental Age; n.r.: not reported; RCT: Randomized Controlled Trial; UCLA: University of California: Los Angeles

Table S4. Summary of EIBI predictors of positive outcome in studies excluded due to a patient recruitment age range beyond 48 months.

Study	Predictors of better outcome	Improved functions correlated with predictors	Non-predictors
[1]Dimian et al., 2021	• Younger age at intake.<br>• Higher IQ.	• Higher odds of being enrolled in regular (vs. special) classes.<br>• Lower probability of needing special education services at school.	None reported.
[2,3]Eikeseth et al., 2002/2007	• Higher intake IQ.	• Higher post-treatment IQ and language skills and changes in language (but not IQ) scores.<br>• Higher 3-year-follow-up IQ and adaptive behaviors (VABS, except for Social domain).	• Age at intake.
[4]Eikeseth et al., 2012	None found.		• Age at intake.<br>• Adaptive behaviors.<br>• Maladaptive behaviors.<br>• Autism severity symptoms.
[5]Eldevik et al., 2006	• Higher intake IQ.<br>• Higher language skills at intake.	• Post-treatment IQ.<br>• Adaptive behaviors.<br>• Non-verbal IQ.<br>• Language skills.	• Age at intake.<br>• Non-verbal IQ.<br>• Adaptive behaviors
[6]Eriksson et al., 2013	• Not having a medical/genetic condition (including epilepsy).<br>• No history of regression.<br>• Older age at intake.	• Adaptive behaviors (VABS).	None reported.
[7]Flanagan et al., 2012	• Younger age at intake.<br>• Adaptive behaviors.*	• Post-treatment IQ.	Autism severity symptoms.

Study	Predictors of better outcome	Improved functions correlated with predictors	Non-predictors
[8]Frazier et al., 2021	• Younger age at intake.<br>• Lower autistic symptoms severity.<br>• Higher verbal and non-verbal cognitive functions.	• Post-treatment language skills.	None reported.
[9]Goin-Kochel et al., 2007	• Overall pre-treatment functioning.<br>• Responsiveness to treatment within the first 6 months.<br>• Younger age at intake.	• Overall post-treatment functioning.<br>• Improved adaptive behaviors.	None reported.
[10]Granpeesheh et al., 2009	• Younger age at intake.	• Number of monthly mastered behavioral objectives.	None reported.
[11]Harris & Handleman, 2000	• Younger age at intake.<br>• Higher IQ at intake.	• Higher odds of being enrolled in regular (vs. special) education class.	• Autism severity symptoms
[12]Klintwall and Eikeseth, 2012	• Older age at intake.<br>• Number of socially mediated (vs. automatic) stimuli that functioned as reinforcers.	• Better learning rate.	• Adaptive behaviors
[13]Lewon and Ghezzi 2021	• 3-months improvement in expressive language skills and receptive language skills.	• Improved adaptive behaviors.<br>• Improved severity symptoms (except for stereotyped behaviors).	• Age at intake.<br>• Adaptive behaviors.<br>• Autism symptoms severity.<br>• Non-verbal imitation
[14]Luiselli et al., 2000	None found (except for treatment intensity).		• Age at intake.

Study	Predictors of better outcome	Improved functions correlated with predictors	Non-predictors
[15,16]Magiati et al., 2007; 2011	• Higher IQ at intake.<br>• Receptive language.<br>• Adaptive behaviors.<br>• Autism symptoms severity.	• Better overall post-treatment gains.<br>• Except for severity symptoms, these variables predicted treatment outcome at 5-years follow-up.	• Age at intake.
[17]Perry et al., 2011	• Higher IQ at intake.<br>• Younger age at intake.<br>• Higher adaptive behaviors at intake.<br>• Autism symptoms severity at intake.	• Post-treatment adaptive behaviors.<br>• Post-treatment IQ.<br>• Post-treatment smptoms severity.	None reported.
[18]Préfontaine et al., 2022	• Level of pre-treatment impairment profile.	Children with pre-treatment moderate to severe impairment profile continued to progress overall one year after the end of EIBI in conceptual and social domain, while children with pre-treatment mild impairment profile continued to progress in practical domain.	None reported.
[19]Rivard et al., 2019	• Higher IQ at intake.<br>• Autism severity symptoms at intake.	• 1-year follow-up IQ and adaptive behaviors.<br>• 1-year follow-up severity symptoms.	• Adaptive behaviors.
[20]Smith et al., 2015	• Higher pre-treatment functioning.<br>• Younger age at intake.<br>• MSEL DQ at intake.<br>• Social engagement.	• Overall better post-treatment and 1-year follow-upfunction.<br>• Improved post-treatment and 1-year follow-up DQ and adaptive behavior.<br>• Improved post-treatment symptoms severity.	• Sensory-motor rituals.

Study	Predictors of better outcome	Improved functions correlated with predictors	Non-predictors
[21]Stoelb et al., 2004	• Having no dysmorphic physical features nor history of regression.<br>• Older age at intake.	• Better outcome at 6 and 12 months.<br>• Better post-treatment language skills.	• Overall pretreatment functioning.<br>• MRI results.<br>• Head circumference.<br>• History of seizures.<br>• Sleep problems.<br>• Gender.<br>• “Complex”** autism
[22]Virués-Ortega et al., 2013	• Younger age at intake.<br>• Pre-intervention functioning.	• Gross motor skills, receptive language, self-care skills and social behaviors.<br>• Fine motor skills, prewriting skills, cognitive abilities and expressive language.	None reported
[23]Waters et al., 2018	Younger age was at treatment start.	• Non-verbal IQ/DQ<br>• Adaptive behaviors.	None reported
[24]Weiss, 1999	Rate of learning.	• Improved adaptive behaviors and symptoms severity.	None reported

* Analysis performed on a subgroup of children: n=61 from intervention group vs. n=61 from comparison group.
**All participants who exhibited abnormal MRI results, microcephaly or physical dysmorphology were classified as complex autism cases.
ABLLS: Assessment of Basic Language and Learning Skills; ADI-R: Autism Diagnostic Interview-Revised; ADOS: Autism Diagnostic Observation Schedule; DQ: Developmental Quotient; EIBI: Early Intensive Behavioral Intervention; GARS: Gilliam Autism Rating Scale; IQ: Intellectual Quotient; MSEL: Mullen Scales of Early Learning; TAU: Treatment as Usual; VABS: Vineland Adaptive Behavior Scale.

Table S5. Summary of ESDM studies excluded from our systematic review due to a patient recruitment age range beyond 48 months: sample characteristics.

Study	Cases				Controls			
	N (M:F)	Age at intake in months (mean)	Diagnosis	Excluding criteria	Control Intervention	N (M:F)	Age at intake in months (mean)	Diagnosis
[25]Fulton et al., 2014	38 (35:3)	39-64 (52)	DSM-IV-TR	Neurological disorders; Significant hearing, vision, motor or physical impairments	---	---	---	---
[26]Geoffrey et al., 2019	19 (15:4)	22-50 (35)	DSM-5; ADOS-2	Severe medical conditions	---	---	---	---
[27]Laister et al., 2021	56 (51:5)	29-60 (42)	DSM-IV; ADOS-2	Nonverbal DA < 12 months; Diagnosis > 48 months of age	---	---	---	---
[28]Robain et al., 2020	22 (22:0)	20-60 (31)	DSM-5; ADOS-2	Neurodevelopmental disorders of known genetic etiology; Severe medical condition	CT	38 (38:0)	20-60 (40)	ASD
[29]Sinai-Gavrilov et al., 2020	26 (20:6)	33-57 (44)	DSM-5; ADOS-2	n.r.	MDI	25 (22:3)	33-57 (45)	ASD
[30]Vivanti et al., 2013	21 (20:1)	22-58 (38)	ADOS	Severe medical conditions; Significant vision, hearing, motor, or physical problems	---	---	---	---
[31]Vivanti et al., 2014*	27 (23:4)	18-60 (40)	ADOS	CA < 18 or > 60 months	CT	30 (27:3)	18-60 (42)	ASD

*Part of the sample was included in Vivanti et al., 2013.
ADOS: Autism Diagnostic Observation Schedule; ASD: Autism Spectrum Disorder; CA: Chronological Age; CT: Community Therapy; DSM: Diagnostic and Statistical Manual of Mental Disorders; MDI: Multidisciplinary Developmental Intervention; n.r.: not reported.

Table S6. Summary of ESDM studies excluded from our systematic review due to a patient recruitment age range beyond 48 months: intervention characteristics.

Study	Country	Study design	Setting	Intensity	Duration
[25]Fulton et al., 2014	Australia	One group pretest-posttest design	Centre-based (GS)	17-22 hours/week	12 months
[26]Geoffrey et al., 2019	France	One group pretest-posttest design	Clinic-based; Home/Preschool/Nursery	4-10 (m=8) hours/week	10 months
[27]Laister et al., 2021	Austria	One group pretest-posttest design	Centre/Home/Preschool	4,5 hours/week	12 months
[28]Robain et al., 2020	Switzerland	Case-control trial	Not reported	20 hours/week	12 months
[29]Sinai-Gavrilov et al., 2020	Israel	Case-control trial	Preschool-based (GS)	~44 hours/week	8 months
[30]Vivanti et al., 2013	Australia	One group pretest-posttest design	Centre-based (GS)*	15-25 hours/week	12 months
[31]Vivanti et al., 2014	Australia	Case-control trial	Centre-based (GS)*	15-25 hours/week	12 months

GS: Group Setting.

Table S7. Summary of ESDM predictors of positive outcome in studies excluded due to a patient recruitment age range beyond 48 months.

Study	Predictors of better outcome	Improved functions correlated with predictors	Non-predictors
[25]Fulton et al., 2014	• Higher IQ/DQ.<br>• Lower ASD symptoms at intake.<br>• VABS Daily Living Skills.<br>• VABS Motor Skills/MSEL fine motor<br>• VABS Communication<br>• MSEL Receptive and Expressive Language	• Greater overall post-treatment improvement.	• VABS Socialization.<br>• VABS Internalizing behaviors
[26]Geoffrey et al., 2019	• Fine motor skills.<br>• Non-verbal DQ.	• Post-treatment DQ.	• Age at intake.<br>• Autism severity symptoms.
[27]Laister et al., 2021	• Better social-communication behaviors.<br>• Better verbal and non-verbal DQ.	• Overall verbal and non-verbal gains post-treatment.	• Age at intake.<br>• Multilingualism.<br>• Autism severity symptoms.
[28]Robain et al., 2020	• Preference for social stimuli.<br>• Younger age at intake.<br>• Lower DQ at baseline.<br>• Lower maladaptive behaviors.	• Post-treatment DQ.	None reported.
[29]Sinai-Gavrilov et al., 2020	• Lower symptoms severity.<br>• Higher adaptive functioning.<br>• Higher MSEL DQ.	• Overall treatment response: gain in MSEL Age Equivalent over pre-treatment DQ	• Age at start.
[30]Vivanti et al., 2013	• Functional use of objects.<br>• Imitation.<br>• Symptoms severity.<br>• Goal understanding.	• Non-verbal skills.<br>• Motor skills.<br>• Language skills.	• Age at intake.<br>• IQ.<br>• Social attention.

Study	Predictors of better outcome	Improved functions correlated with predictors	Non-predictors
[31]Vivanti et al., 2014	• Lower symptoms severity.	• Post-treatment symptoms severity.	• Age at intake.<br>• IQ.<br>• Language skills.<br>• Adaptive behaviors

ADOS: Autism Diagnostic Observation Schedule; ASD: Autism Spectrum Disorder; DQ: Developmental Quotient; IQ: Intellectual Quotient; MSEL: Mullen Scales of Early Learning; VABS: Vineland Adaptive Behavior Scale.

Table S8. Complete list of references, predictors, statistics used in each original article, single p-value either provided in each original article or derived from the published statistics (in red), combined using Fisher's statistics to obtain the cumulative p-values presented in Table 7 of our published manuscript.

Supplementary References

[1] Dimian, A. F.; Symons, F. J.; Wolff, J. J. Delay to Early Intensive Behavioral Intervention and Educational Outcomes for a Medicaid-Enrolled Cohort of Children with Autism. *J Autism Dev Disord* **2021**, *51* (4), 1054–1066. https://doi.org/10.1007/s10803-020-04586-1.

[2] Eikeseth, S.; Smith, T.; Jahr, E.; Eldevik, S. Intensive Behavioral Treatment at School for 4- to 7-Year-Old Children with Autism: A 1-Year Comparison Controlled Study. *Behav Modif* **2002**, *26* (1), 49–68. https://doi.org/10.1177/0145445502026001004.

[3] Eikeseth, S.; Smith, T.; Jahr, E.; Eldevik, S. Outcome for Children with Autism Who Began Intensive Behavioral Treatment Between Ages 4 and 7: A Comparison Controlled Study. *Behav Modif* **2007**, *31* (3), 264–278. https://doi.org/10.1177/0145445506291396.

[4] Eikeseth, S.; Klintwall, L.; Jahr, E.; Karlsson, P. Outcome for Children with Autism Receiving Early and Intensive Behavioral Intervention in Mainstream Preschool and Kindergarten Settings. *Research in Autism Spectrum Disorders* **2012**, *6* (2), 829–835. https://doi.org/10.1016/j.rasd.2011.09.002.

[5] Eldevik, S.; Eikeseth, S.; Jahr, E.; Smith, T. Effects of Low-Intensity Behavioral Treatment for Children with Autism and Mental Retardation. *J Autism Dev Disord* **2006**, *36* (2), 211–224. https://doi.org/10.1007/s10803-005-0058-x.

[6] Eriksson, M. A.; Westerlund, J.; Hedvall, Å.; Åmark, P.; Gillberg, C.; Fernell, E. Medical Conditions Affect the Outcome of Early Intervention in Preschool Children with Autism Spectrum Disorders. *Eur Child Adolesc Psychiatry* **2013**, *22* (1), 23–33. https://doi.org/10.1007/s00787-012-0312-7.

[7] Flanagan, H. E.; Perry, A.; Freeman, N. L. Effectiveness of Large-Scale Community-Based Intensive Behavioral Intervention: A Waitlist Comparison Study Exploring Outcomes and Predictors. *Research in Autism Spectrum Disorders* **2012**, *6* (2), 673–682. https://doi.org/10.1016/j.rasd.2011.09.011.

[8] Frazier, T. W.; Klingemier, E. W.; Anderson, C. J.; Gengoux, G. W.; Youngstrom, E. A.; Hardan, A. Y. A Longitudinal Study of Language Trajectories and Treatment Outcomes of Early Intensive Behavioral Intervention for Autism. *J Autism Dev Disord* **2021**, *51* (12), 4534–4550. https://doi.org/10.1007/s10803-021-04900-5.

[9] Goin-Kochel, R. P.; Myers, B. J.; Hendricks, D. R.; Carr, S. E.; Wiley, S. B. Early Responsiveness to Intensive Behavioural Intervention Predicts Outcomes Among Preschool

Children with Autism. *International Journal of Disability, Development and Education* **2007**, *54* (2), 151–175. https://doi.org/10.1080/10349120701330404.

[10] Granpeesheh, D.; Dixon, D. R.; Tarbox, J.; Kaplan, A. M.; Wilke, A. E. The Effects of Age and Treatment Intensity on Behavioral Intervention Outcomes for Children with Autism Spectrum Disorders. *Research in Autism Spectrum Disorders* **2009**, *3* (4), 1014–1022. https://doi.org/10.1016/j.rasd.2009.06.007.

[11] Harris, S. L.; Handleman, J. S. Age and IQ at Intake as Predictors of Placement for Young Children with Autism: A Four- to Six-Year Follow-Up. *Journal of Autism and Developmental Disorders* **2000**, *30* (2), 137–142. https://doi.org/10.1023/A:1005459606120.

[12] Klintwall, L.; Eikeseth, S. Number and Controllability of Reinforcers as Predictors of Individual Outcome for Children with Autism Receiving Early and Intensive Behavioral Intervention: A Preliminary Study. *Research in Autism Spectrum Disorders* **2012**, *6* (1), 493–499. https://doi.org/10.1016/j.rasd.2011.07.009.

[13] Lewon, A. B.; Ghezzi, P. M. An Evaluation of the Early Learning Measure as a Predictor of Outcomes in Early Intensive Behavioral Intervention. *Behavioral Interventions* **2021**, *36* (2), 388–406. https://doi.org/10.1002/bin.1768.

[14] Luiselli, J. K.; Cannon, B. O.; Ellis, J. T.; Sisson, R. W. Home-Based Behavioral Intervention for Young Children with Autism/Pervasive Developmental Disorder. A Preliminary Evaluation of Outcome in Relation to Child Age and Intensity of Service Delivery. *Autism* **2000**, *4* (4), 426–438. https://doi.org/10.1177/1362361300004004007.

[15] Magiati, I.; Charman, T.; Howlin, P. A Two-Year Prospective Follow-up Study of Community-Based Early Intensive Behavioural Intervention and Specialist Nursery Provision for Children with Autism Spectrum Disorders. *J Child Psychol & Psychiat* **2007**, *48* (8), 803–812. https://doi.org/10.1111/j.1469-7610.2007.01756.x.

[16] Magiati, I.; Moss, J.; Charman, T.; Howlin, P. Patterns of Change in Children with Autism Spectrum Disorders Who Received Community Based Comprehensive Interventions in Their Pre-School Years: A Seven Year Follow-up Study. *Research in Autism Spectrum Disorders* **2011**, *5* (3), 1016–1027. https://doi.org/10.1016/j.rasd.2010.11.007.

[17] Perry, A.; Cummings, A.; Geier, J. D.; Freeman, N. L.; Hughes, S.; Managhan, T.; Reitzel, J.-A.; Williams, J. Predictors of Outcome for Children Receiving Intensive Behavioral Intervention

in a Large, Community-Based Program. *Research in Autism Spectrum Disorders* **2011**, *5* (1), 592–603. https://doi.org/10.1016/j.rasd.2010.07.003.

[18] Préfontaine, I.; Morizot, J.; Lanovaz, M. J.; Rivard, M. A Person-Centered Perspective on Differential Efficacy of Early Behavioral Intervention in Children with Autism: A Latent Profile Analysis. *Research in Autism Spectrum Disorders* **2022**, *97*, 102017. https://doi.org/10.1016/j.rasd.2022.102017.

[19] Rivard, M.; Morin, M.; Mello, C.; Terroux, A.; Mercier, C. Follow-Up of Children With Autism Spectrum Disorder 1 Year After Early Behavioral Intervention. *Behav Modif* **2019**, *43* (4), 490–517. https://doi.org/10.1177/0145445518773692.

[20] Smith, T.; Klorman, R.; Mruzek, D. W. Predicting Outcome of Community-Based Early Intensive Behavioral Intervention for Children with Autism. *J Abnorm Child Psychol* **2015**, *43* (7), 1271–1282. https://doi.org/10.1007/s10802-015-0002-2.

[21] Stoelb, M.; Yarnal, R.; Miles, J.; Takahashi, T. N.; Farmer, J. E.; McCathren, R. B. Predicting Responsiveness to Treatment of Children With Autism: A Retrospective Study of the Importance of Physical Dysmorphology. *Focus Autism Other Dev Disabl* **2004**, *19* (2), 66–77. https://doi.org/10.1177/10883576040190020101.

[22] Virues-Ortega, J.; Rodríguez, V.; Yu, C. T. Prediction of Treatment Outcomes and Longitudinal Analysis in Children with Autism Undergoing Intensive Behavioral Intervention. *International Journal of Clinical and Health Psychology* **2013**, *13* (2), 91–100. https://doi.org/10.1016/S1697-2600(13)70012-7.

[23] Waters, C. F.; Amerine Dickens, M.; Thurston, S. W.; Lu, X.; Smith, T. Sustainability of Early Intensive Behavioral Intervention for Children With Autism Spectrum Disorder in a Community Setting. *Behav Modif* **2018**, *1* (24), 3–26. https://doi.org/10.1177/0145445518786463.

[24] Weiss, M. J. Differential Rates of Skill Acquisition and Outcomes of Early Intensive Behavioral Intervention for Autism. *Behav. Intervent.* **1999**, *14* (1), 3–22. https://doi.org/10.1002/(SICI)1099-078X(199901/03)14:1<3::AID-BIN25>3.0.CO;2-F.

[25] Fulton, E.; Eapen, V.; Crnčec, R.; Walter, A.; Rogers, S. Reducing Maladaptive Behaviors in Preschool-Aged Children with Autism Spectrum Disorder Using the Early Start Denver Model. *Front. Pediatr.* **2014**, *2* (40), 1–10. https://doi.org/10.3389/fped.2014.00040.

[26] Geoffray, M.-M.; Laboratory Health Services and Performance Research (HESPER), Lyon, France; Denis, A.; Hospices Civils de Lyon, Pôle de Santé Publique, Lyon, France; Mengarelli,

F.; Peter, C.; Gallifet, N.; Beaujeard, V.; Department for Child and Adolescent psychiatry, Centre Hospitalier le Vinatier, Bron, France; Grosmaitre, C. J.; Malo, V.; Department for Child and Adolescent psychiatry, Centre Hospitalier de Saint Jean de Dieu, Lyon, France; Grisi, S.; Georgieff, N.; Department for Child and Adolescent psychiatry, Centre Hospitalier le Vinatier, Bron, France; Magnificat, S.; Department for Child and Adolescent psychiatry, Centre Hospitalier de Saint Jean de Dieu, Lyon, France; Touzet, S.; Laboratory Health Services and Performance Research (HESPER), Lyon, France; Hospices Civils de Lyon, Pôle de Santé Publique, Lyon, France. Using ESDM 12 Hours per Week in Children with Autism Spectrum Disorder: Feasibility and Results of an Observational Study. *Psychiat Danub* **2019**, *3* (31), 333–339. https://doi.org/10.24869/psyd.2019.333.

[27] Laister, D.; Stammler, M.; Vivanti, G.; Holzinger, D. Social-Communicative Gestures at Baseline Predict Verbal and Nonverbal Gains for Children with Autism Receiving the Early Start Denver Model. *Autism* **2021**, *25* (6), 1640–1652. https://doi.org/10.1177/1362361321999905.

[28] Robain, F.; Franchini, M.; Kojovic, N.; Wood de Wilde, H.; Schaer, M. Predictors of Treatment Outcome in Preschoolers with Autism Spectrum Disorder: An Observational Study in the Greater Geneva Area, Switzerland. *J Autism Dev Disord* **2020**, *50* (11), 3815–3830. https://doi.org/10.1007/s10803-020-04430-6.

[29] Sinai-Gavrilov, Y.; Gev, T.; Mor-Snir, I.; Vivanti, G.; Golan, O. Integrating the Early Start Denver Model into Israeli Community Autism Spectrum Disorder Preschools: Effectiveness and Treatment Response Predictors. *Autism* **2020**, *24* (8), 2081–2093. https://doi.org/10.1177/1362361320934221.

[30] Vivanti, G.; Dissanayake, C.; Zierhut, C.; Rogers, S. J.; Victorian ASELCC Team. Brief Report: Predictors of Outcomes in the Early Start Denver Model Delivered in a Group Setting. *J Autism Dev Disord* **2013**, *43* (7), 1717–1724. https://doi.org/10.1007/s10803-012-1705-7.

[31] Vivanti, G.; Paynter, J.; Duncan, E.; Fothergill, H.; Dissanayake, C.; Rogers, S. J.; the Victorian ASELCC Team. Effectiveness and Feasibility of the Early Start Denver Model Implemented in a Group-Based Community Childcare Setting. *J Autism Dev Disord* **2014**, *44* (12), 3140–3153. https://doi.org/10.1007/s10803-014-2168-9.
